# Potential interactions between antineoplastic agents and medicines used to treat Covid-19

**DOI:** 10.1177/10781552211040494

**Published:** 2021-10-12

**Authors:** Mario Jorge Sobreira da Silva, Claudia Garcia Serpa Osorio-de-Castro, Rafael Duarte Paes, Christopher Lucas Negrete, Elizangela Eugênio, Elaine Lazzaroni Moraes, Annemeri Livinalli

**Affiliations:** 1 37908 National Cancer Institute of Brazil, Brazil; 2 42499 Sergio Arouca National School of Public Health, Brazil; 3 434749 Oncoclinicas of Brazil, Brazil; 4 42522 Sírio-Libanês Hospital, Brazil; 5 67754 AMO Clinic, Brazil; 6 CF Onco, Brazil

**Keywords:** Covid-19, drug–drug interactions, antineoplastic agents

## Abstract

**Introduction:**

Cancer patients with Covid-19 are exposed to treatment combinations that can potentially result in interactions that adversely affect patient outcomes. This study aimed to identify potential drug–drug interactions between antineoplastic agents and medicines used to treat Covid-19.

**Methods:**

We conducted a search for potential interactions between 201 antineoplastic agents and 26 medicines used to treat Covid-19 on the Lexicomp^®^ and Micromedex^®^ databases. The following data were extracted: interaction severity (“major” and “contraindicated”) and interaction effects (pharmacokinetic and pharmacodynamic). We also sought to identify the therapeutic indication of the antineoplastic drugs involved in the potential drug–drug interactions.

**Results:**

A total of 388 “major” or “contraindicated” drug–drug interactions were detected. Eight drugs or combinations (baricitinib, lopinavir/ritonavir, atazanavir, darunavir, azithromycin, chloroquine, hydroxychloroquine, and sirolimus) accounted for 91.5% of these interactions. The class of antineoplastic agents with the greatest potential for interaction was tyrosine kinase inhibitors (accounting for 46.4% of all interactions). The findings show that atazanavir, baricitinib, and lopinavir/ritonavir can affect the treatment of all common types of cancer. The most common pharmacokinetic effect of the potential drug–drug interactions was increased plasma concentration of the antineoplastic medicine (39.4%).

**Conclusions:**

Covid-19 is a recent disease and pharmacological interventions are undergoing constant modification. This study identified a considerable number of potential drug–drug interactions. In view of the vulnerability of patients with cancer, it is vital that health professionals carefully assess the risks and benefits of drug combinations.

## Introduction

According to the World Health Organization’s International Agency for Research on Cancer (IARC), it is estimated that there will be more than 25 million new cases of cancer and 16.5 cancer deaths worldwide by 2040.^1^

Cancer patients are frequently older (≥60 years) and tend to have one or more key comorbidities. Moreover, they tend to experience a decline in immune function, making them more susceptible to respiratory diseases such as pneumonia, especially in the case of hematologic malignancies.^2–5^

Cancer patients have been defined as a risk group for severe Covid-19 ever since the start of the coronavirus pandemic in 2020.^6^ There are no recommendations for deviations from standard care for patients with cancer,^7,8^ who should be offered adequate treatment, including chemotherapy. However, a recently published systematic review and meta-analysis shows that patients on active chemotherapy may be at a higher risk of death from Covid-19.^9^ Cancer patients with Covid-19 are exposed to treatment combinations that can potentially result in loco interactions that adversely affect patient outcomes.

Various types of chemotherapy regimens involving the use of different combinations of injectable and oral drugs cause side effects. The efficacy and safety of currently available treatments for Covid-19 have yet to be fully evaluated. This is particularly due to the evolution of experimental therapies and a growing body of evidence of the multisystemic effects of SARS-CoV-2.^10-12^ A cohort study involving cancer patients with Covid-19 identified 49 different treatment patterns, showing that the most commonly used medicines were hydroxychloroquine, azithromycin, remdesivir, high-dose corticosteroids, and tocilizumab, taken alone and in combination.^6^

The increasing number of new cancer cases, substantial heterogeneity in cancer types, patient vulnerability, a diverse range of chemotherapy protocols, high transmissibility of SARS-CoV-2, and a wide variety of treatments currently adopted around the world are cause for concern.^4^

The aim of this study was to identify potential interactions between antineoplastic agents and medicines used to treat Covid-19 on the Lexicomp^®^ and Micromedex^®^ databases in order to contribute to the therapeutic management of cancer patients on chemotherapy.

## Method

We conducted a descriptive study with the aim of analyzing potential drug–drug interactions (DDIs) between Covid-19-related treatments (trials or clinical experience) and drugs used to treat cancer.

The medicines used to treat Covid-19 were selected from a list of 34 drugs published by the American Society of Health-System Pharmacists (ASHP) on May 1st 2020.^13^ After analyzing the list, it was decided to exclude medicines used for both cancer and Covid-19 (dexamethasone, hydrocortisone, and methilprednisolone), as well as those with no evidence to support use in the treatment of Covid-19 (ibuprofen, HMG-CoA reductase inhibitors, nelfinavir, saquinavir, and tipranavir), resulting in a final sample of 26 medicines (ascorbic acid, albuterol, alteplase, anakinra, atazanavir, azithromycin, baloxavir, baricitinib, chloroquine, colchicine, darunavir, epoprostenol, favipiravir, heparin, hydroxychloroquine, immunoglobulin, indomethacin, ivermectin, lopinavir/ritonavir, nitazoxanide, nitric oxide, oseltamivir, remdesivir, ruxolitinib, sirolimus, and tocilizumab).

The list of drugs used to treat cancer was obtained from the Brazilian Manual of Clinical Oncology.^14^ A total of 228 medicines were identified. Medicines administered by topical, inhalation, ocular, and otic routes were excluded, resulting in a final sample of 201 antineoplastic agents. In this regard, antineoplastic agents administered by the topical^15^ and ocular (intravitreal) routes^16^ show low absorption, while the inhalation and otic routes are not commonly used for the administration of anticancer drugs.

A search for potential interactions between medicines from both groups was conducted using the Lexicomp^®^ and Micromedex^®^ databases. These widely used databases are constantly updated and offer high-performing tools for analyzing anticancer drug interactions.^17^

We used the International Nonproprietary Name (INN) of the selected medicines to detect potential interactions. The following information was extracted from the databases: interaction severity and interaction effects.

With regard to severity, interactions with the following ratings were selected: Lexicomp^®18^—“D. Consider Therapy Modification” or “X. Avoid Combination”; Micromedex^®19^—“Major” or “Contraindicated”. The ratings “D. Consider Therapy Modification” and “major”, and “X. Avoid Combination” and “contraindicated” were considered equivalent for the purposes of this study. When the interaction rating differed between the databases (for example “X” on Lexicomp^®^ and “major” on Micromedex^®^), we considered the most severe rating. Strength of Recommendation and Strength of evidence were not considered.

The data were collected in June 2020 and compiled using a Microsoft Excel^®^ spreadsheet.

Interaction effects were grouped into pharmacokinetic effects and pharmacodynamic effects. We also sought to identify the therapeutic indications of the antineoplastic agents involved in the potential DDI in order to detect the types of cancer most affected by interaction effects. Cancer types were grouped according to the affected system or organ as follows: hematologic malignancies (lymphoid leukemia, myeloid leukemia, lymphoma, multiple myeloma, and myelodysplastic syndromes) and solid tumors (head and neck, gastrointestinal, genitourinary, gynecologic, breast, melanoma and skin, lung and soft tissue).

The medicines identified as having potential DDIs were categorized based on the WHO’s Anatomical Therapeutic Chemical (ATC) classification system.^20^ For presentation purposes, the antineoplastic agents were grouped into therapeutic classes using the third level of the ATC system.

## Results

**Figure 1.**
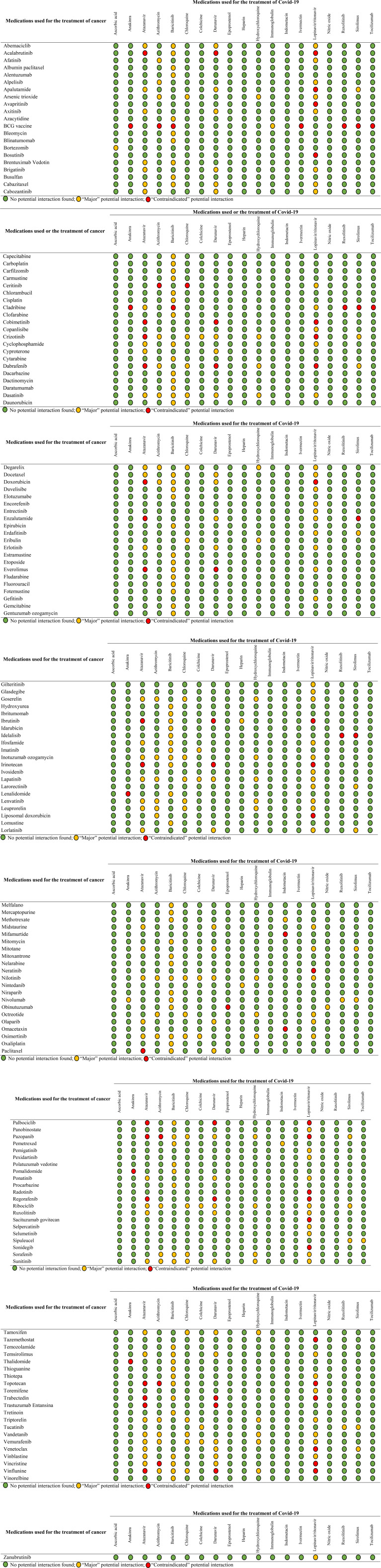
Potential interactions between antineoplastics and medications used for the treatment of Covid-19 by interaction severity.

We compiled 5,526 potential combinations between the two drug groups and detected 388 potential DDIs rated as “major” or “contraindicated”. Figure 1 shows the potential DDIs according to severity.

**Figure 2.**
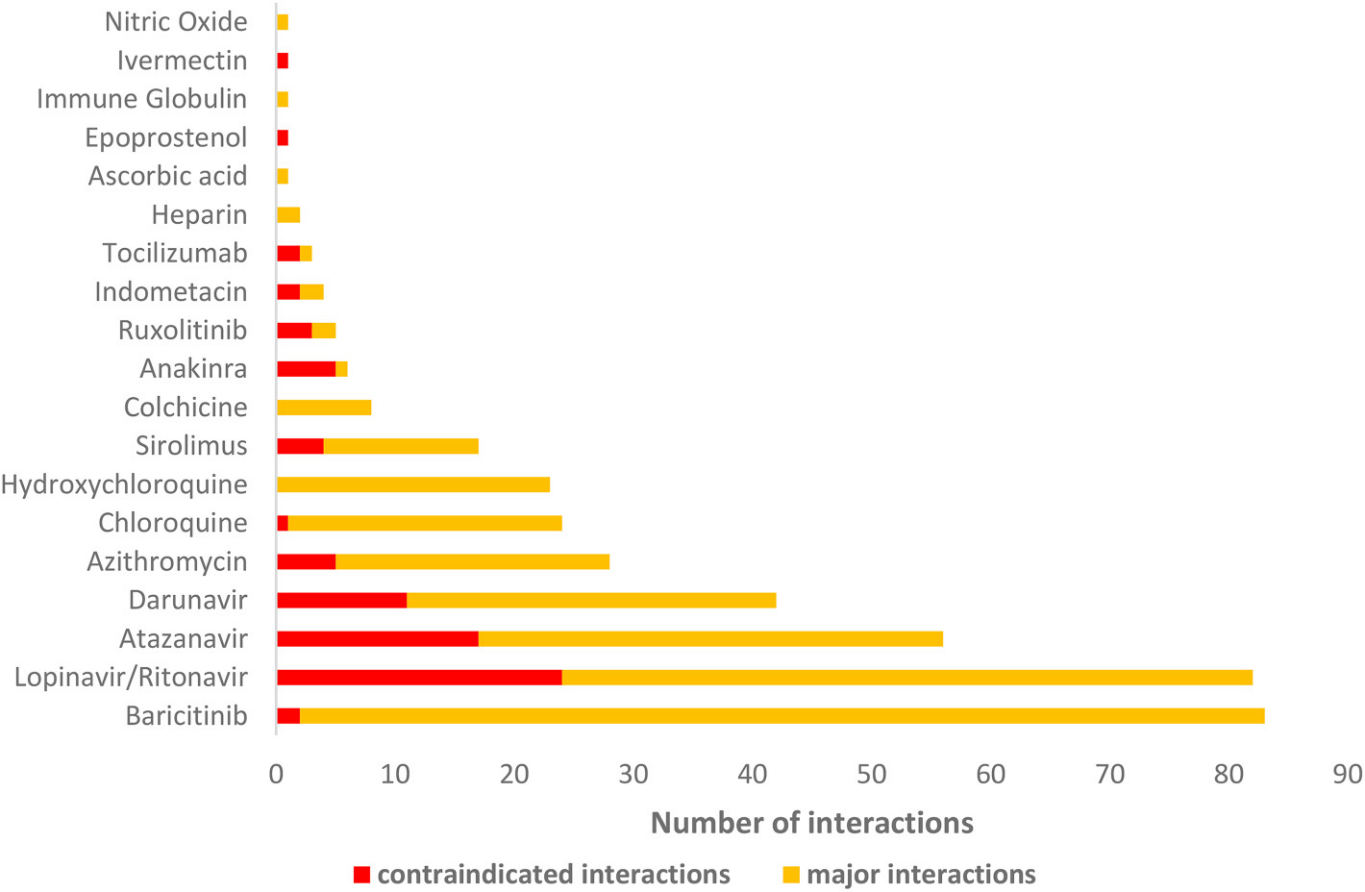
Number of “contraindicated” and “major” interactions between antineoplastic agents and medicines used to treat Covid-19.

Nineteen of the 26 drugs used to treat Covid-19 had potential DDIs (Figure 2). Eight drugs or combinations (baricitinib, lopinavir/ritonavir, atazanavir, darunavir, azithromycin, chloroquine, hydroxychloroquine, and sirolimus) accounted for 91.5% of the “contraindicated” or “major” DDIs. Baricitinib showed the highest risk of interaction, with 83 “contraindicated” or “major” interactions (21.4%), followed by lopinavir/ritonavir, with 82 (21.1%).

The class of antineoplastic agents that showed the highest number of interactions with medicines used to treat Covid-19 was tyrosine kinase inhibitors (TKIs, 46.4%), followed by other antineoplastic agents (13.9%) and plant alkaloids (10.8%) (Table 1).

**Table 1.** Severity of potential interactions between antineoplastic agents and medicines used to treat Covid-19 according to the third level of the Anatomical Therapeutic Chemical (ATC) classification system.

Pharmacological group (ATC code)	Severity of potential drug–drug interactions			
	Contraindicated	Major	Total	%
Tyrosine kinase inhibitors (L01E)	33	147	180	46.4
Other antineoplastic agents (L01X)	6	48	54	13.9
Plant alkaloids and other natural products (L01C)	15	27	42	10.8
Antimetabolites (L01B)	5	16	21	5.4
Hormone antagonists and related agents (L02B)	3	15	18	4.6
Alkylating agents (L01A)	0	16	16	4.1
Hormones and related agents (L02A)	0	15	15	3.9
Cytotoxic antibiotics and related substances (L01D)	3	11	14	3.6
Immunostimulants (L03A)	8	3	11	2.8
Immunosuppressants (L04A)	3	4	7	1.8
Hypothalamic Hormones (H01C)	0	4	4	1.0
Unclassified agents^a^	2	1	3	0.8
Antiandrogens	0	2	2	0.5
Other therapeutic radiopharmaceuticals (V10X)	0	1	1	0.3

ATC: Anatomical Therapeutic Chemical.

^a^ Unclassified agents: Sacituzumab govitecan, Selpercatinib, and Tazemetostat.

Based on the pharmacologic class and chemotherapy regimens, we listed the potential interactions between antineoplastic agents and medicines used to treat Covid-19 according to the type of malignancy (Table 2). The findings show that atazanavir, baricitinib, and lopinavir/ritonavir can affect the treatment of all common types of cancer. The malignancies that showed the highest number of DDIs were: hematologic malignancies—lymphoid leukemia (10.0%) and lymphoma (9.2%); solid tumors—genitourinary (14.5%), lung (13.3%), and breast (12.2%) cancer.

**Table 2.** Potential interactions between antineoplastic agents and medicines used to treat Covid-19 according to type of malignancy.

Medicines used to treat Covid-19	Hematologic malignancies					Solid tumors							
	Lymphoid leukemia	Myeloid leukemia	Lymphoma	Multiple myeloma	Myelodysplastic syndromes	Head and neck	Gastrointestinal	Genitourinary	Gynecologic	Breast	Melanoma and skin cancer	Lung	Soft tissue
Ascorbic acid	−	−	1	−	−	−	−	−	−	−	−	−	−
Anakinra	1	−	2	1	1	1	1	1	−	−	1	1	1
Atazanavir	8	4	7	1	1	3	9	15	7	14	4	13	6
Azithromycin	5	2	3	2	1	3	6	8	5	5	3	9	3
Baricitinib	17	11	24	8	4	8	17	19	13	16	9	16	12
Chloroquine	3	1	−	−	−	−	3	4	1	4	1	4	1
Colchicine	2	−	−	−	1	−	−	−	−	1	1	2	−
Darunavir	5	3	4	1	−	2	9	13	3	11	4	11	5
Epoprostenol	1	−	−	−	−	−	−	−	−	−	−	−	−
Heparin	1	−	1	−	−	−	−	−	−	−	−	1	−
Hydroxychloroquine	3	1	−	−	−	−	3	4	1	4	1	3	1
Immunoglobulin	−	−	−	−	−	−	−	−	−	−	−	−	−
Indomethacin	1	1	1	−	−	1	−	−	−	1	−	1	2
Ivermectin	−	−	−	−	−	−	−	1	−	−	−	−	−
Lopinavir/ritonavir	9	10	11	3	1	3	11	16	8	16	6	17	8
Nitric oxide	1	−	−	−	−	−	−	−	−	−	−	−	−
Ruxolitinib	2	−	1	−	−	1	1	1	−	1	1	1	−
Sirolimus	2	1	2	−	−	1	2	6	−	2	2	4	−
Tocilizumab	1	−	−	−	−	−	−	2	−	−	−	−	−
Total	62	34	57	16	9	23	62	90	38	76	33	83	39

A relevant pharmacokinetic effect (increased plasma drug concentration) was identified in 264 of the DDIs: 164 DDIs (39.4%) can result in increased plasma concentration of the medicine used to treat Covid-19; and 100 DDIs (24.0%) can result in increased plasma concentration of the antineoplastic agent. With regard to pharmacodynamic effects, increased risk of changes in cardiac parameters was identified in 98 (23.6%) of the potential DDIs (Table 3).

**Table 3.** Effects of the potential interactions between antineoplastic agents and medicines used to treat Covid-19.

Effects of the potential drug–drug interactions	Number	%
Increased plasma concentration of the antineoplastic agent	164	39.4
Increased plasma concentration of the medicine used to treat Covid-19	100	24.0
Increased risk of changes in cardiac parameters	98	23.6
Others	27	6.5
Reduced plasma concentration of the antineoplastic agent	17	4.1
Reduced plasma concentration of the medication used to treat Covid-19	10	2.4

## Discussion

Cancer patients and the health professionals who care for them are facing unprecedented challenges in these times of Covid-19, with evidence suggesting that cancer patients are especially vulnerable to the disease.^21^

The literature highlights the complexity of potential interactions between drugs used to treat Covid-19 and antineoplastic agents.^22^ Cancer patients are, per se, at increased risk of DDIs.^22^ Since only a limited number of studies provide robust evidence of the nature of these interactions, taking into account the disease morbidity rate,^9^ it is important to alert oncology practitioners to the potential risks to patients beyond those posed by the disease.

Our findings show that, when used in different combinations, 201 antineoplastic agents and 26 medicines used to treat Covid-19 resulted in 388 potentially severe (“major” or “contraindicated”) DDIs. This is a considerable number, bearing in mind the relative intensity of treatment for Covid-19 in debilitated patients with impaired body functions, who may require high doses of different drugs, thus increasing the risk of interactions.^6,23^

Among the investigational drugs used to treat Covid-19, baricitinib showed the greatest potential for interaction with the antineoplastic agents analyzed by this study. Based on the findings of the ACTT-2 study,^24^ in November 2020, the Food and Drug Administration (FDA) granted authorization for the emergency use of baricitinib in combination with remdesivir to treat Covid-19.^23,25,26^ Most of the baricitinib interactions involve targeted therapy drugs, especially TKIs used for the treatment of hematologic malignancies (dasatinib, imatinib, idelalisib, and nilotinib). Baricitinib also has major interactions with multikinase inhibitors (pazopanib, sunitinib, and sorafenib) and cyclin-dependent kinase inhibitors (abemaciclib and palbociclib) which are commonly used on malignant solid tumors. These interactions are explained by the fact that TKIs potently inhibit the hepatic uptake transporters OATP1B1 and OATP1B3,^27^ one of the substrates of baricitinib, leading to decreased renal clearance and an increase in the area under the concentration time curve (AUC).^28^

The findings of the current study show that lopinavir/ritonavir had the largest number of “contraindicated” interactions. Lopinavir/ritonavir is used to treat the human immunodeficiency virus (HIV) in combination with other antiretroviral drugs. However, there is still no evidence of efficacy of the drug against Covid-19.^29^ Lopinavir and ritonavir are potent inhibitors of CYP3A4,^30,31^ which is the most abundant cytochrome P450 isoform in the human body and responsible for the metabolism of many drugs,^30^ significantly affecting TKIs, particularly those used for the treatment of advanced lung cancer with activating mutations, malign hematologic neoplasms and breast cancer.^32^

The findings show that atazanavir has one of the highest numbers of “contraindicated” and “major” DDI interactions when used in combination with antineoplastic agents. The drug is a protease inhibitor used in combination with other antiretroviral agents for the treatment of HIV.^33^ Its indication as an experimental drug for the treatment of Covid-19 appears to be based on data reported by a study showing the potency of binding atazanavir with 2019-nCoV 3C-like proteinase.^34^ Atazanavir is a substrate and moderate inhibitor of cytochromes P450, particularly CYP3A4, which is an inhibitor of uridine diphosphate gluronosyltransferase 1A1 (UGT1A1), potent inhibitor of hepatic uptake transporters OAT and inhibitor of the breast cancer resistance protein (BCRP), which facilitates interactions with various antineoplastic agents, potentially leading to an increased risk of toxicities.^35^

Darunavir is another antiretroviral protease inhibitor indicated for the treatment of HIV.^36^ Together with atazanavir, darunavir was developed to combat drug resistance mutations, mainly by increasing the number of polar interactions with the main atoms in the HIV protease chain. Studies using computational molecular modeling indicate that both darunavir and atazanavir are promising drugs for the treatment of Covid-19, as SARS-COV-2 is also part of the family of RNA viruses.^37^ Darunavir is a substrate and strong inhibitor of CYP3A4, inhibitor of CYP2D6, and substrate and inducer of P-glycoprotein (P-gp), which explains its potential to interact with alkylating agents, antimetabolites, taxanes, topoisomerase inhibitors, and various TKIs.^35^

With regard to cancer treatment, the findings show that protease inhibitors such as atazanavir and darunavir are a class of medicines with high risk of interactions with antineoplastic agents in both cytotoxic and targeted molecular therapy. Medicines in this class should therefore be indicated with caution for the experimental treatment of Covid-19 in patients with cancer.

Based on the preliminary findings of in vitro studies demonstrating its immunomodulatory properties, azithromycin is now considered for the clinical management of Covid-19.^26,38,39^ However, clinical studies show that it cannot be safely concluded that the drug provides benefit to patients.^40^ Azithromycin was associated with 23 “major” antineoplastic interactions. Most of these interactions are related to TKIs, especially those used to treat advanced lung cancer with activating mutations (EGFR—afatinib and osimertinib; ALK—crizotinib; KRAS—dabrafenib and vemurafenib),^41^ breast cancer (lapatinib and ribociclib), and other types of solid malignant tumors involving multikinase inhibitors (lenvatinib, sorafenib, sunitinib, and vandetanib).^42^ The interaction between azithromycin and antineoplastic agents appears to be related to pharmacodynamic mechanisms, characterized by an increased risk of changes in cardiac parameters.^43^

Chloroquine and hydroxychloroquine emerged as prominent drugs for the clinical management of Covid-19 at the beginning of the pandemic.^44^ However, there is currently no clinical evidence corroborating their use.^12,45^ Despite the relative safety of these drugs in the treatment of autoimmune diseases and malaria, they are associated with severe cardiotoxic effects,^46^ especially when used in combination with other medicines that increase the possibility of interactions. Their use in combination TKIs results in kinetic effects due to the inhibition of P-gp.^47,48^ We identified 23 “major” hydroxychloroquine interactions, more than half of which are related to TKIs.

Sirolimus, which has been used to treat Covid-19,^49^ had 17 interactions (4 “contraindicated” and 13 “major”) with antineoplastic agents. Sirolimus is a substrate of CYP3A4 and P-gp^50^ with high potential for interaction with TKIs, especially those used for the treatment of advanced lung cancer with activating mutations (crizotinib, lorlatinib, and dabrafenib).^51^

The only drug investigated by this study to have obtained approval for the treatment of Covid-19 from the FDA,^52^ European Medicines Agency (EMA),^53^ and Brazil’s National Health Surveillance Agency (ANVISA)^54^ is the antiviral agent remdesivir. We did not find any potential interactions between remdesivir and antineoplastic agents on the databases. However, this “lack” of interaction should be treated with caution, as new medicines generally have few studies investigating DDIs.

The class of antineoplastic agents that showed the greatest potential for interaction with medicines used to treat Covid-19 was TKIs, with 180 “major” or “contraindicated” interactions. Most of the potential DDIs for this group were related to the risk of increased plasma concentration of the TKI, followed by increased heart-related risks, such as QT interval prolongation and increased plasma concentration of the medicine used to treat Covid-19. The use of TKIs with other drugs that reduce absorption or induce metabolism can result in sub-therapeutic levels of the drugs and bring about a decrease in TKI effect.^55^ In contrast, drugs that inhibit the metabolism of TKIs can cause supra-therapeutic drug levels and toxicity.^56^

Most of the potential DDIs involve possible pharmacokinetic effects, which include both the potential toxicity of antineoplastic agents and drugs used to treat Covid-19 and the potentiation of these effects. It is also important to stress that cancer patients may have serious system impairment, including reduced renal and hepatic function,^22^ making them more susceptible to pharmacokinetic effects caused by potential DDIs.

Our findings show that 71.4% of the potential DDIs are related to the combined use of medicines used to treat Covid-19 and antineoplastic agents used for solid tumors, especially genitourinary, lung and breast cancer. This may be related to the large number of TKIs indicated for malignant diseases,^57^ given that this class of medicines has greater potential for interactions. Grivas et al.^58^ pointed to an association between recent cytotoxic chemotherapy and higher Covid-19 severity and disease mortality.

One of the limitations of the current study is that it was not possible to detect all potential DDIs. In this regard, 5,138 of the combinations between antineoplastic agents and medicines used to treat Covid-19 compiled by this study did not result in potential DDIs. Thereof two possible reasons for this: the combination does not have an interaction; or the interaction had not been included on the databases by the time the data was collected.

Another aspect is the ‘novelty’ of possible interactions between drugs used to treat Covid-19 and antineoplastic agents. Covid-19 is a recent disease and pharmacological interventions are undergoing constant modification as new evidence arises from research and direct data from medication use.

However, both positive and negative results provide information for oncologists, who are ultimately responsible for therapeutic decision-making. It is worth highlighting that many medicines have lost credibility during the fight against Covid-19 and evidence of the absence of drug benefits is gaining currency in clinical practice. In cases of unclear or absent benefits, the risk outweighs the benefits, meaning that the possibility of interaction should be subject to scrutiny and caution.
